# Creation of independently controllable multiple focal spots from segmented Pancharatnam-Berry phases

**DOI:** 10.1038/s41598-018-28186-3

**Published:** 2018-06-29

**Authors:** Peng Li, Xuyue Guo, Shuxia Qi, Lei Han, Yi Zhang, Sheng Liu, Yu Li, Jianlin Zhao

**Affiliations:** MOE Key Laboratory of Material Physics and Chemistry under Extraordinary Conditions, Shaanxi Key Laboratory of Optical information Technology, School of Science, Northwestern Polytechnical University, Xi’an, 710129 China

## Abstract

Recently, based on space-variant Pancharatnam-Berry (PB) phases, various flat devices allowing abrupt changes of beam parameters have been predicted and demonstrated to implement intriguing manipulation on spin states in three dimensions, including the efficient generation of vector beams, spin Hall effect of light and light-guiding confinement, and so on. Here, we report on the construction of independently controllable multiple focal spots with different inhomogeneous polarization states by utilizing segmented PB phases. Combining the phase shift approach with PB phases, we engineer fan-shaped segmented PB phases and encode them onto two spin components that compose a hybrid polarized vector beam in a modified common-path interferometer system. Experimental results demonstrate that the fan-shaped segmented PB phase enables the flexible manipulation of focal number, array structure and polarization state of each focal spot. Furthermore, we demonstrate that this fan-shaped approach enables to flexibly tailor the polarization state and the spin angular momentum distribution of a tightly focused field, which have potential applications in optical manipulation, tailored optical response and imaging etc.

## Introduction

The Pancharatnam-Berry (PB) phase elements involving spin-orbit interaction for controlling and manipulating electromagnetic wave behaviors is currently an active research area^1–9^. Various optical elements with specific architectures and designed PB phases, including patterned subwavelength grating^10,11^, liquid crystal devices^12,13^ and metasurfaces^4,14^, have been successively reported and demonstrated being efficient for creating beams with inhomogeneous polarization state, that is, the vector beams^15^. On the other hand, PB phase supports an effect approach to manipulate the propagation dynamics of two spin states, due to the corresponding relationship between phase gradient structure in position space and wave vector in momentum space. Utilizing tailored PB phases, some conceptual and practical implications, such as the spin Hall effect of light^3,6^, light-guiding confinement^5^, multiwavelength achromatic focusing^16^, etc. have been deeply developed. Additionally, owing to the geometric flexibility, the PB phase structure has been further expanded to complex functions and the nonlinear realm, to achieve geometrical field^17^ and functional effect^18^. The modulation on PB phases not only reveals a serial of intriguing phenomena, more importantly, it promotes the development of spin-optics, and further pushes award the applications, e.g., this remarkable prospect of spin-dependent separation in holographic imaging has excited new research directions^19,20^.

Recently, the vector beams possessing inhomogeneous polarization states have been intensively researched. Considerable tightly focused fields with specific intensity, polarization and phase structures have been proposed toward promising applications for super-resolution imaging, optical trapping, and light micro-fabrication^15^. However, with the developed requirement for optical parallel operation and highly efficient optical processing, single focused spot has been unable to meet the application efficiency of focusing field and to enhance the parallelism of the optical system. Consequently, researchers gradually turned attention to the multifocal array with independent controllability, involving the generation and modulation mechanism in free space^21–24^ and optical waveguide^25^. The multifocal arrays with inhomogeneous polarization states and orbital angular momenta (OAMs) have presented application potentials in multifocal microscopic imaging, optical trapping and manipulation^26–29^. Remarkably, the PB phase-based multiple focus generation scheme has also been exploited by using segmented metasurface^30^, exhibiting unique potential in the simultaneous control of the spin angular momentum (SAM) and OAM of focal field.

In this article, we report on the creation and control of multiple focal spots from fan-shaped segmented PB phases. According to the common phase shift approach, we engineer fan-shaped segmented PB phases and encode them onto two spin components that compose a hybrid polarized vector beam in a modified common-path interferometer system. Experimental results demonstrate that the fan-segmented PB phases enable the flexible manipulation of focal number, array structure and polarization state of each focus. Furthermore, we propose ingenious focal fields by numerically analyzing the fan-shaped segmented vector fields based on vector diffraction theory. These results can be extended to steer the polarization state and angular momentum distribution of tightly focused field and create topological structure such as Möbius ring^31^.

## Theory Principle

To construct multiple focal spots or focal array, we employ the displacement theory of Fourier transform, that the phase shift in spatial domain corresponds to a displacement in the spectral domain (momentum space). According to the Fourier transform of lens, for a incident beam denoted as *E*_0_(*x*, *y*), the corresponding field in spectral domain can be expressed as

1 $${\bf{E}}({x}_{f}-{\rm{\Delta }}{x}_{f},{y}_{f}-{\rm{\Delta }}{y}_{f})= {\mathcal F} \{\exp [\,-\,i2\pi ({f}_{x}x+{f}_{y}y)]{{\bf{E}}}_{0}(x,y)\},$$

where **E**_0_ is the incident electric field, ${\mathcal F} (\,\cdot \,)$ denotes the Fourier transform, ${f}_{x}={\rm{\Delta }}{x}_{f}/\lambda f$ and ${f}_{y}={\rm{\Delta }}{y}_{f}/\lambda f$ are the spatial spectra in the *x* and *y* directions, ${\rm{\Delta }}{x}_{f}$ and ${\rm{\Delta }}{y}_{f}$ are the displacements in the spectral domain. Here we denote the phase shift as ${\rm{\Phi }}(r,\varphi )=2\pi ({f}_{x}x+{f}_{y}y)$, with *x* = *r*cos(*ϕ*) and *y* = *r*sin(*ϕ*), respectively.

**Figure 1:**
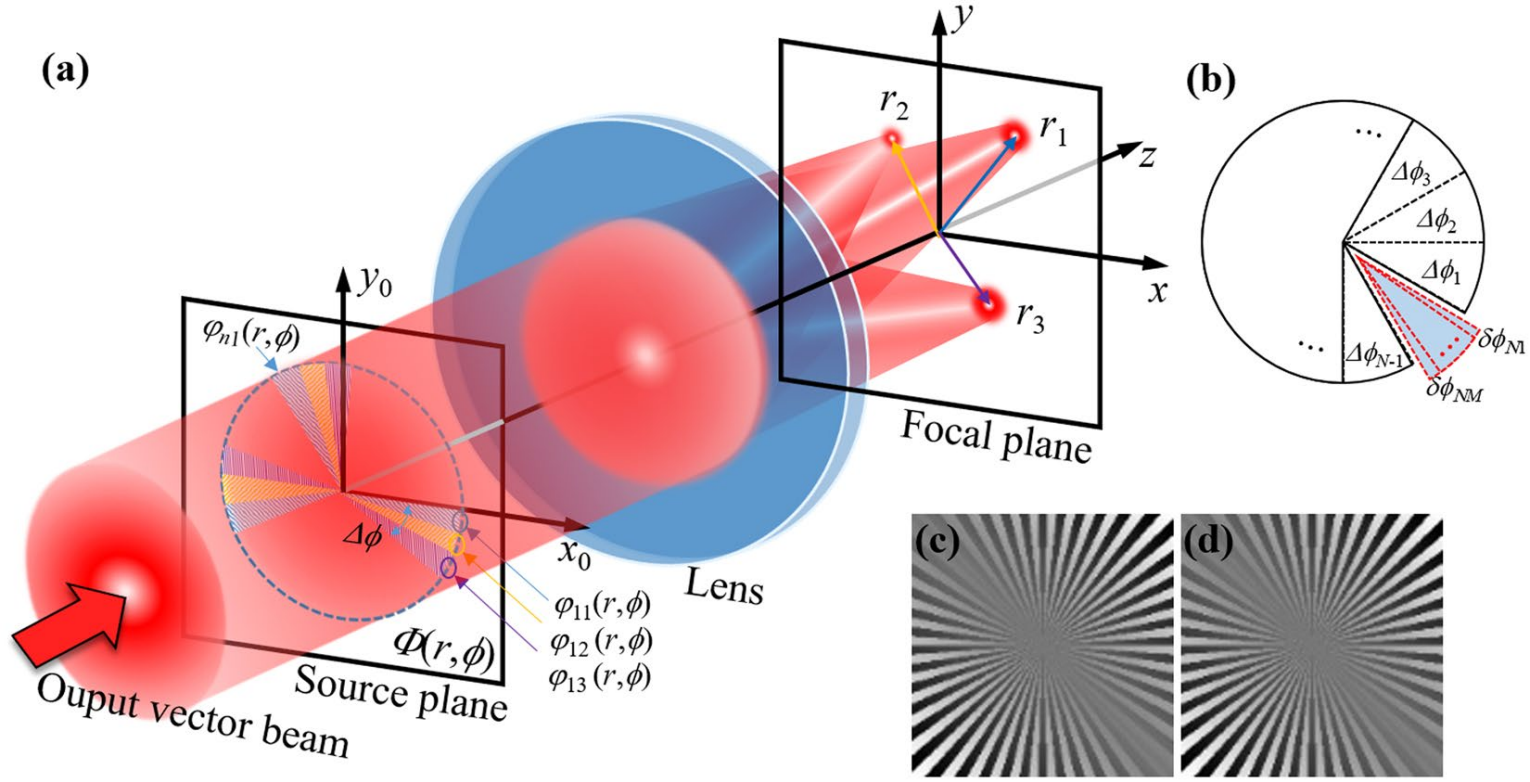
Schematic diagram showing the construction of multiple focal spots from a vector beam based on PB phases. (**a**) Focusing model; (**b**) segmenting principle for fan-shaped PB phases; (**c**) and (**d**) PB phase examples attached on two spin states. The output vector beam here refers to the output of the modulating device which will be discussed with the help of Fig. 5.

According to Eq. (1), to separate the focal field into several discrete spots and keep the cylindrical intensity profiles, we divide the pupil plane into *N* identical fan-shaped segments with angle width ${\rm{\Delta }}\varphi =2\pi /N$, and further divide each segment into *M* subsegments with angle width $\delta \varphi =2\pi /(NM)$, as shown in Fig. 1(b). Here, *M* is the number of focus^23^.

Next, to construct vector field, we decompose the light field into two spin components, i.e., $${{\bf{E}}}_{0}(r,\varphi )\,=$$$${{\bf{E}}}_{0}[a\,\exp (\,-\,i{\phi }_{0}){{\bf{e}}}_{R}+b\,\exp (i{\phi }_{0}){{\bf{e}}}_{L}]$$, where *φ*_0_ is a constant phase, *a* and *b* are the normalized amplitudes with relationship *a*^2^ + *b*^2^ = 1, $${{\bf{e}}}_{R,L}$$ denote the right- and left-handed circular polarizations, respectively^32,33^. Then, utilizing a polarization conversion system, we independently control the polarization conversion process of two spin components. After the polarization conversion system, two spin components get different geometric phases that are independent on the dynamic processes, i.e., the Pancharatnam-Berry (PB) phases^34^. Supposing that the incident beam has a linear polarization, i.e., $$a=b=1/\sqrt{2}$$, and the PB phases attached on two spin components are Φ = ±*lϕ*, the superposed field thus can be expressed as $${{\bf{E}}}_{0}(r,\varphi )={{\bf{E}}}_{0}[\exp (\,-\,il\varphi -i{\phi }_{0}){{\bf{e}}}_{R}+\exp (il\varphi +i{\phi }_{0}){{\bf{e}}}_{L}]/\sqrt{2}$$^35^. All of the nonessential factors have been absorbed into the envelop profile *E*_0_. For such a case, the output field after the polarization conversion system is a *l* th-order vector beam.

In this principle, we engineer fan-shaped segmented PB phases that can independently control the polarization state of each focus. A generalized phase structures can be expressed as^34^

2 $${{\rm{\Phi }}}_{R,L}(r,\varphi )=\sum _{n=1}^{N}\,\sum _{m=1}^{M}\pm {\phi }_{nm}(r,\varphi )+{{\bf{k}}}_{nm}\cdot {\bf{r}},$$

where, *φ*_*nm*_(*r*, *ϕ*) = *l*_*m*_*ϕ* + *φ*_0*m*_ is the segmented PB phase, ${{\bf{k}}}_{nm}=2\pi {r}_{m}[\,\cos ({\varphi }_{m}){{\bf{e}}}_{x}+\,\sin ({\varphi }_{m}){{\bf{e}}}_{y}]$ denotes the wavevector, with (*r*_*m*_, *ϕ*_*m*_) corresponds to the central position of the *m* th focus. The focusing model is schematically illustrated in Fig. 1. After the polarization conversion system, the output beam possessing hybrid polarization state is divided and focused into *M* focal spots, under the combining modulation of focusing lens and PB phases. Meanwhile, a pair of spin components attaching with opposite states construct a vector focal, of which the polarization state is dependent on the topological charge *l*_*m*_ and constant phase *φ*_0*m*_. For the sake of simplify, we depict the focal position in cylindrical coordinate, that is, replacing Δ*f*_*x*_ and Δ*f*_*y*_ by *r*_*m*_ and *ϕ*_*m*_. From the focusing model and PB phases described in Eq. (2), we find that the parameters Δ*f*_*x*_ and Δ*f*_*y*_, as well as *l*_*m*_ and *φ*_0*m*_ enable the possibility of independently steering each focus, including the transverse position and polarization state.

## Results and Discussions

**Figure 2:**
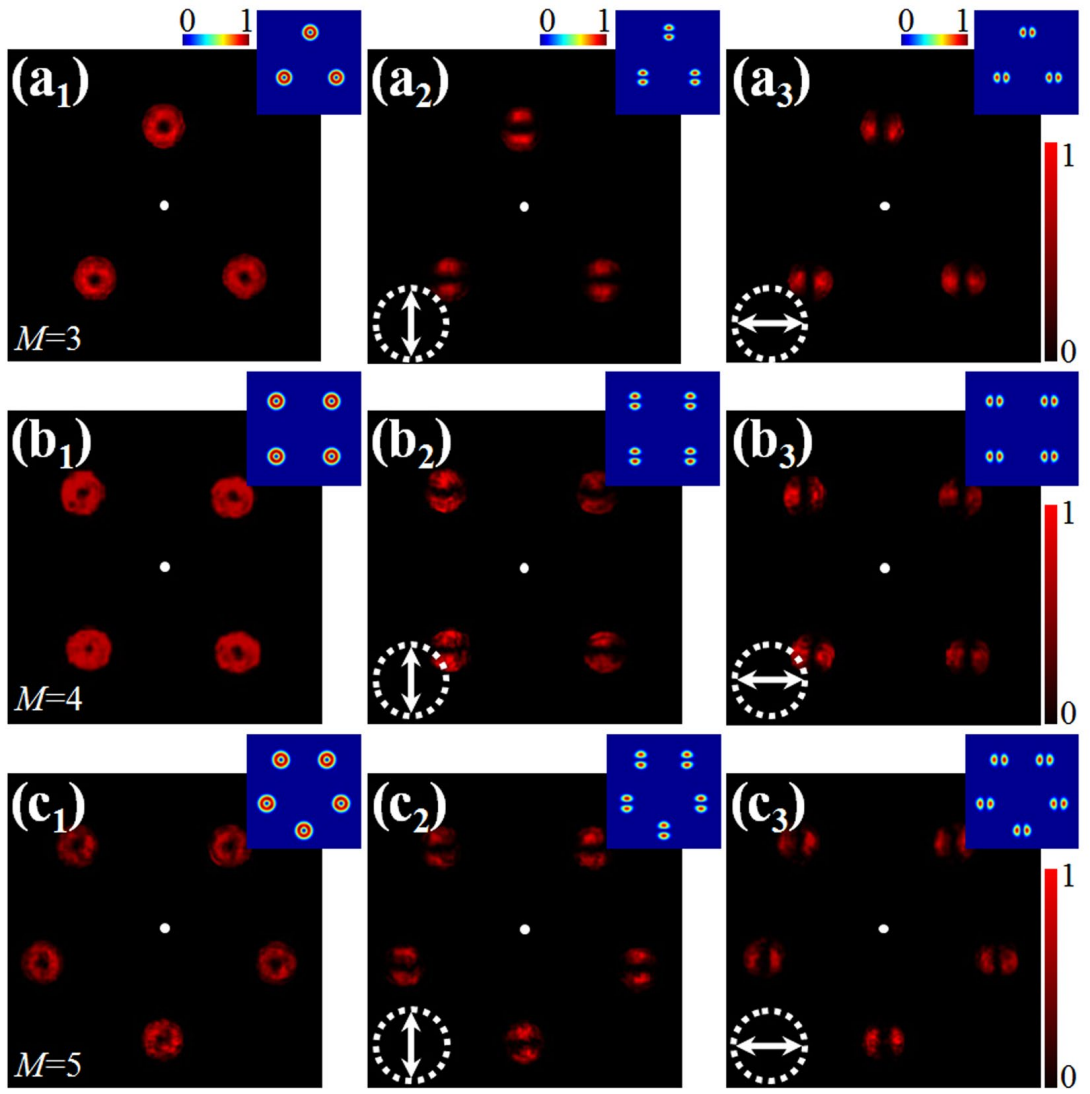
Intensity distributions of focal fields with different focus numbers. (**a**) *M* = 3; (**b**) *M* = 4; (**c**) *M* = 5. The left column corresponds to the total intensity, the middle and right columns correspond to the intensity distributions after a linear polarizer, of which the polarization orientation is depicted as the white arrows. Insets: Numerical calculated intensity distributions according to vector diffraction integral theory. The dimension for all images is 2.7 mm × 2.7 mm.

We first engineer PB phases to construct rotationally symmetric focal field, namely, the focal fields with multiple identical focal spots, to verify our theory principle. Fig. 2 displays the intensity distribution of focal fields for the cases of *M* = 3, 4 and 5, and *N* = 40. The total intensity distributions for different symmetries are shown in the left column, and the middle and right columns show the intensity distributions after a vertical and horizontal polarizer, respectively. The insets are corresponding numerical simulation results according to the vector diffraction integral theory^36,37^. In experiment, in order to directly present the focal field, we employ a Lens of *f* = 10 cm to focus the vector field. That is, *NA* = 0.15. As a result, the *z*-component is negligible. It can be seen that, these focal spots present identical intensity and polarization distributions, indicating the possibility of this approach.

**Figure 3:**
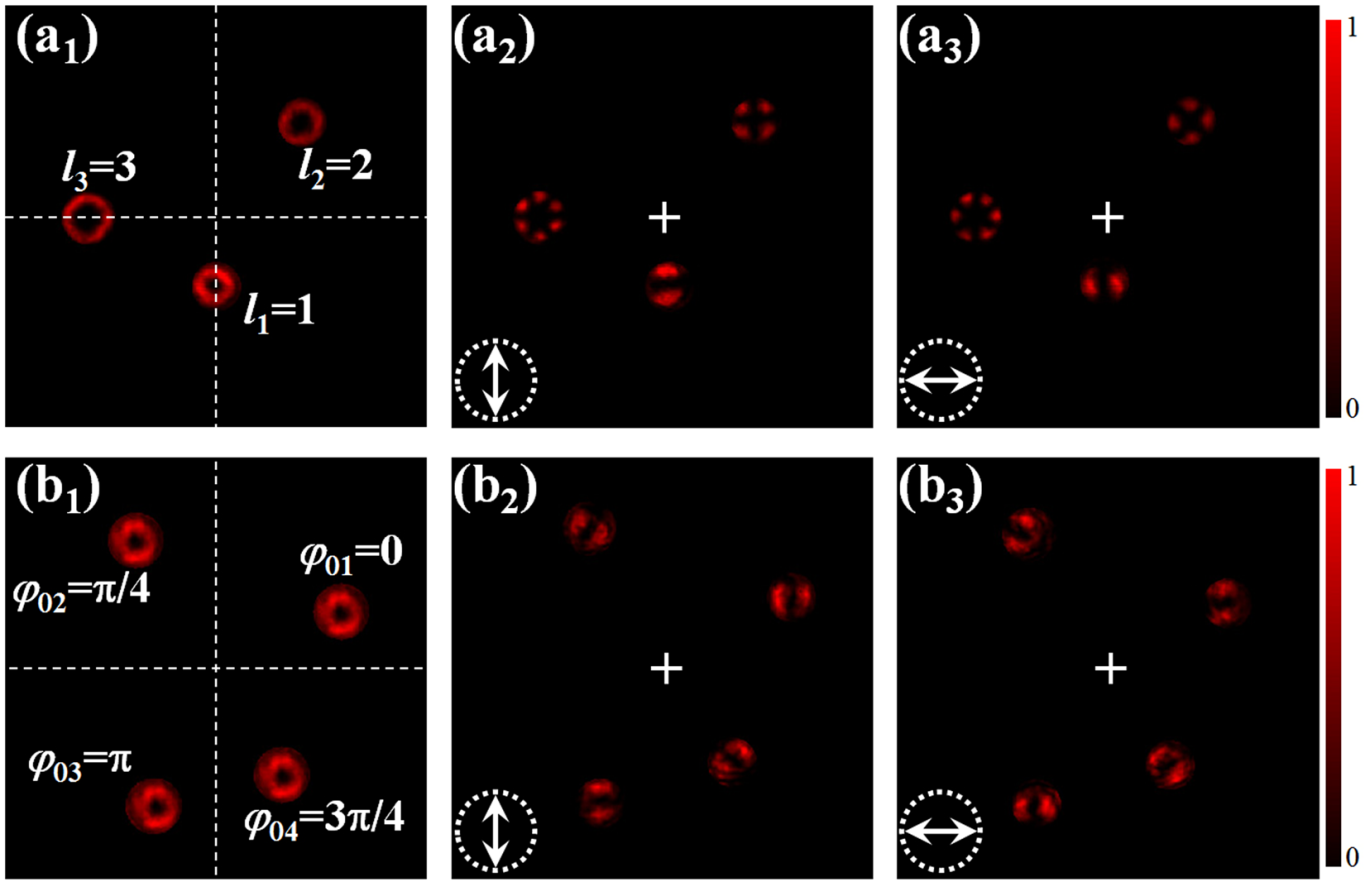
Intensity distributions of the focal fields with (**a**) three and (**b**) four independent vector focal spots. Left column: total intensity distributions; middle and right columns: intensity distributions after passing through a linear polarizer orientated along the arrows shown in figures, respectively.

Next, we demonstrate the independent control of multiple focal spots based on the steering of PB phases. Here, we first create three independent focal spots with different polarization orders, i.e., *l*_1_ = 1, *l*_2_ = 2, *l*_3_ = 3. Meanwhile, we set the positional parameters as *r*_1_ = *r*_2_/2 = *r*_3_/2 and *ϕ*_1_ = *ϕ*_2_/4 = −*ϕ*_3_ = *π*/4. Fig. 3(a) shows the focal intensity distributions and the corresponding results after passing through a horizonal or vertical polarizer. As expected, three vector spots with different polarization orders are observed in the focal plane, besides, the transverse displacements are consistent with the input parameters, that the second- and third-order vector spots have two times displacement than the first-order one, and their angle positions are tunable.

Figure 3(b) shows the case of four first-order vector focal spots, i.e., *M* = 4. Likewise, we set these four spots have different transverse displacements, and variously initial phases *φ*_0*m*_. In such a special case, *φ*_0*m*_ = *mπ*/4, therefore, the polarization state of four spots orderly rotate *π*/4, as shown in the Fig. 3(b1) and (b2). These experimental results indicate the independent control of multiple focal spots according to the fan-shaped segmented PB phases. Besides, the fan-shaped segment is advisable for high-order vector fields.

The above experimental results indicate the possibility and flexibility of controlling multiple focus of a vector beam in the focal plane based on fan-shaped segmented PB phases. However, It is should be clear that the quality of focal spot is strongly depend on the spot and segment numbers, i.e., parameters *N* and *M*^23^. For a case that has larger spot number, to ensure the quality of spots and reduce the effect of angular discontinuity, the parameter *N* also should have a larger value, thus each subsegment has a smaller angular width. This is challenging for experimental implementation, confined by the pixel resolution of spatial light modulator (SLM), the low resolution of central region of PB phases lead to the inhomogeneous intensity of the generated vector field, and further affect the focal intensity patterns. In practice, for the cases of $M > 5$, although the influence induced by the central region can be reduced by enlarging the beam size and adding spatial filter, the experimentally produced focal spot quality will manifestly decrease.

**Figure 4:**
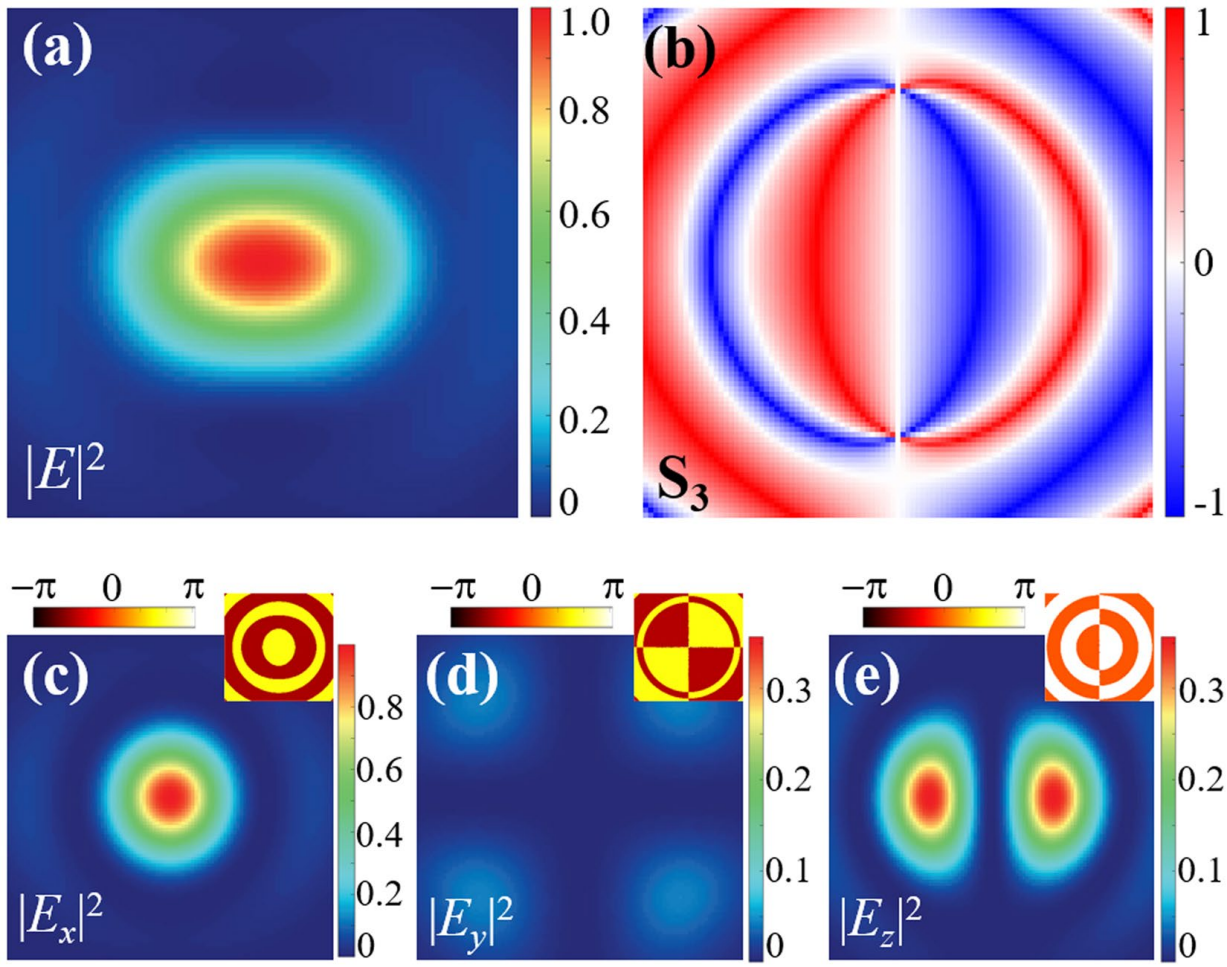
Focal field constructed by three fan-shaped constituent vector beams. (**a**) Total intensity distribution; (**b**) transverse SAM density *S*_*y*_; (**c**–**e**) intensity and phase (insets) distributions of three components.

Nevertheless, it is noteworthy that, PB phase supports simultaneously steering the polarization and phase structures of the generated vector fields^14^, and the constructed vector fields carrying nonzero OAM occur obvious polarization conversion in focusing process^38,39^. Therefore, the superposition of multiple focal spots with distinct polarization and phase structures can further create ingenious focal fields that have desirable polarization structures, even geometrical angular momentum distributions. Figure 4 shows a tailored focal field that is created from the superposition of three focal spots generated from tightly focusing of three fan-shaped constituent beams with diverse polarization and phase structures and angular width. The parameters correspond to three fan-shaped constituent beams are *l*_1*R*_ = 0, *l*_1*L*_ = 2; *l*_2*R*_ = −2, *l*_2*L*_ = 0 and *l*_3*R*_ = *l*_3*L*_ = 1, the angular width ratio is 1:1:2, the numerical aperture of focusing is *NA* = 0.95. For such a special case, we note that the focal field has an oblong intensity pattern. Importantly, from the intensity and phase distributions of three components shown in Fig. 4(c–e), one can see that the *y* component of the focal field is negligible, compared with the other two components. In addition, the *x* and *z* components have constant phase difference of *π*/2. As a result, the focal filed presents transverse SAM along the *y* direction, as the SAM density presented in Fig. 4(b). Such geometrically tailored transverse SAM have been extensively studied in light-matter interaction for extraordinary optical force, unidirectional coupling and tailored optical response^40,41^.

Beyond that, we note that this PB phase scheme supplies another degree of freedom for controlling the focusing, namely, the longitudinal displacement. For instance, three-dimensional focus shift has been realized by engineering spherical phase $\exp (i{\alpha }_{0m}{r}^{2})$ into the PB phases^34^. Likewise, the fan-shaped segmented PB phases also support three-dimensional displacement of each focus. In addition, we note that, this theoretical principle can be expanded to the popular flatness optical elements that present efficient and exact polarization modulation based on PB phase, e.g. metasurface, liquid crystal plate, *et al*. Since then the focus number can be extended to a considerable quantity. On the other hand, most recently, the vector fields with various polarization states have been introduced into the laser fabrication system, to produce polarization-dependent grating elements, such as the surface subwavelength grating on glass^38^. It is conceivable that, this controllable multiple focal spots would promote the operation efficiency, even achieve the processing of multiple grating elements in one step.

## Conclusion

In conclusion, we have introduced and experimentally implemented independently controlling of multiple focal spots with adjustable focal position and polarization state, based on fan-shaped PB phases. Experimental results demonstrate the possibility and flexibility of manipulating the focal number, array and polarization structures. Furthermore, comparing with traditional generation method of multifocal array, this PB phase-based method enables the steering of polarization and phase structures of focused vector field, to further create ingenious focal field that have desirable polarization structures, even geometrical angular momentum distributions. Meanwhile, it can be expanded to the popular flatness optical elements that have efficient and exact polarization modulation based on PB phase, to improve the focus number and focusing quality.

## Methods

### Experimental setup

**Figure 5:**
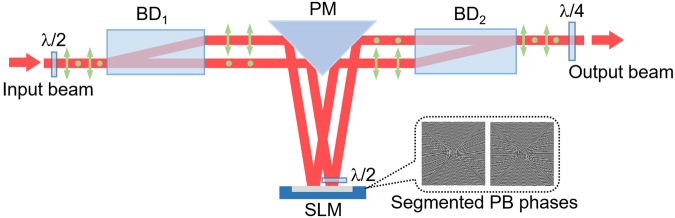
Schematic experimental setup for constructing segmented vector fields. BD: beam displacer; PM: right-angle prism mirror; SLM: spatial light modulator. Inset: Segmented computer-generated holograms containing PB phases encoded on the SLM.

In our experiment, we use a modified common-path interferometry to prepare and control the PB phases. A collimated Gaussian beam with linear polarization from a He-Ne laser at *λ* = 632.8 nm is divided into two orthogonal components via a half-wave plate and beam displacer (Thorlabs, BD40). Then the two components are reflected onto a reflective phase SLM (Holoeye, Leto) by a right-angle prism mirror (PM). Two fan-shaped segmented PB phases, as shown in the insets in Fig. 5, are encoded onto two orthogonally polarized components by the reflective phase SLM. In practice, since the SLM only responses to the horizontally polarized light, another half-wave plate is employed to transform the vertically polarized component into a horizontal one. This component is transformed into the vertical state after passing through the half-wave plate again. Two modulated components then are on-axially synthesized by another beam placer and transformed into opposite spin states by a quarter-wave plate. After that, a 4 f system is employed to filter the segmented vector field with hybrid polarization state, then the output beam is focused by a lens onto a CCD (Phior, SP300).

### Theoretical analysis

In order to elaborately generate multiple focal spots which has independent controllability in the focal plane, it is necessary to appropriately partition the incident beam and then apply different phase modulations. Here, we utilize the vector diffraction integral theory, of which the general focus formula can be expressed as^36,37^

3 $$\begin{array}{rcl}{\bf{E}}({x}_{f},{y}_{f},{z}_{f}) & = & iA\int {\int }_{{\rm{\Omega }}}\,\sin (\theta ){A}_{1}(\theta ,\varphi ){A}_{2}(\theta ,\varphi )[\begin{array}{c}{p}_{x}\\ {p}_{y}\\ {p}_{z}\end{array}]\exp [i{\rm{\Delta }}\alpha (\theta ,\varphi )]\\  &  & \times \,\exp \{ik[r\,\sin \,\theta \,\cos (\varphi -\varphi ^{\prime} )+z\,\cos \,\theta ]\}{\rm{d}}\theta {\rm{d}}\varphi ,\end{array}$$

where, *A* = −*kf*/(2*π*), *A*_1_(*θ*, *ϕ*) is the amplitude of the incident beam, *A*_2_(*θ*, *ϕ*) is a 3 × 3 matrix related to the structure of objective, Δ*α*(*θ*, *ϕ*) is the phase distribution of the incident beam, *p*_*x*_, *p*_*y*_ and *p*_*z*_ is a matrix unit vector about the polarization of incidence light, *θ* is the converge angle given by sin*θ* = *rNA*/(*Rn*_*t*_), *NA* is the numerical aperture of the objective, *n*_*t*_ is the refractive index of the immersion medium, *k* is the wave number, (*r*, *ϕ*) and $(r^{\prime} ,\varphi ^{\prime} )$ are the cylindrical coordinates in the pupil and focal planes, respectively.

In the Debye approximation, the transmitted field **E**_0_ is the plane wave spectrum of the focal field **E**. Hence, the electric field **E** at a point (*x*_*f*_, *y*_*f*_, *z*_*f*_) is obtained by integrating the propagated plane waves, viz

4 $${\bf{E}}({x}_{f},{y}_{f},{z}_{f})=iA\int {\int }_{{\rm{\Omega }}}{{\bf{E}}}_{0}(\theta ,\varphi )\exp [\,-\,i({k}_{x}x+{k}_{y}y+{k}_{z}z)]\,\sin \,\theta \,{\rm{d}}\,\theta \,{\rm{d}}\varphi ,$$

where **E**_0_ is

5 $${{\bf{E}}}_{0}={A}_{1}(\theta ,\varphi ){A}_{2}(\theta ,\varphi )[\begin{array}{c}{p}_{x}\\ {p}_{y}\\ {p}_{z}\end{array}]\exp [i{\rm{\Delta }}\alpha (\theta ,\varphi )],$$

and *k*_*x*_ = −*k*cos*ϕ* sin*θ*, *k*_*y*_ = −*k*sin*ϕ* sin*θ*, *k*_*z*_ = −*k*cos*θ* are the wave vector components, omitting the constant and subsituting spatial frequency in *x* and *y* directions by *f*_*x*_ = −cos*ϕ*sin*θ*/*λ* and *f*_*y*_ = −sin*ϕ*sin*θ*/*λ*, Eq. (4) thus can be rewirttern as

6 $$\begin{array}{rcl}{\bf{E}}({x}_{f},{y}_{f}) & = & {\int }_{0}^{{\theta }_{max}}{\int }_{0}^{2\pi }[{{\bf{E}}}_{0}(\theta ,\varphi )\exp (i{k}_{z}z)/\,\cos \,\theta ]\exp [\,-\,i2\pi ({f}_{x}x+{f}_{y}y)]{\rm{d}}{f}_{x}{\rm{d}}{f}_{y}\\  & = &  {\mathcal F} \{{{\bf{E}}}_{0}(\theta ,\varphi )\exp (i{k}_{z}z)/\,\cos \,\theta \}.\end{array}$$

From Eqs. (5) and (6), it can be seen that the focal field actually is the Fourier transform of the transmitted field. Specially, for a definitive focusing system, i.e., definitive focusing matrix *A*_2_, the parameters *p* and Δ*δ* associated with PB phases are flexible and controllable. This means that engineering PB phases supplies a more fundamental focusing model, meanwhile, reveal a more foreseeable focal field structure.
